# Ecological and Functional Landscape of the Oral Microbiome: A Multi-Site Analysis of Saliva, Dental Plaque and Tongue Coating

**DOI:** 10.3390/microorganisms14010002

**Published:** 2025-12-19

**Authors:** Toru Tamahara, Atsumu Kouketsu, Satoshi Fukase, Pawat Sripodok, Tatsuru Saito, Akiko Ito, Bin Li, Kazuki Kumada, Muneaki Shimada, Masahiro Iikubo, Ritsuko Shimizu, Kensuke Yamauchi, Tsuyoshi Sugiura

**Affiliations:** 1 Tohoku Medical Megabank Organization, Tohoku University, Sendai 980-8573, Miyagi, Japan; ritsuko.shimizu.e8@tohoku.ac.jp; 2 Division of Oral and Maxillofacial Oncology and Surgical Sciences, Department of Disease Management Dentistry, Graduate School of Dentistry, Tohoku University, Sendai 980-8575, Miyagi, Japan; atsumu.kouketsu.c5@tohoku.ac.jp (A.K.); satoshi.hukase.c8@tohoku.ac.jp (S.F.); pawat.sripodok@gmail.com (P.S.); tatsuru.saito.b2@tohoku.ac.jp (T.S.); akiko.ito.b6@tohoku.ac.jp (A.I.); tsuyoshi.sugiura.b2@tohoku.ac.jp (T.S.); 3 Department of Oral and Maxillofacial Pathology, Faculty of Dentistry, Mahidol University, Bangkok 10400, Thailand; 4 Advanced Research Center for Innovations in Next-Generation Medicine, Tohoku University, Sendai 980-8573, Miyagi, Japan; bin.ri.c2@tohoku.ac.jp (B.L.); kazuki.kumada.c7@tohoku.ac.jp (K.K.); muneaki.shimada.b7@tohoku.ac.jp (M.S.); 5 Division of Dental Informatics and Radiology, Graduate School of Dentistry, Tohoku University, Sendai 980-8575, Miyagi, Japan; masahiro.iikubo.c6@tohoku.ac.jp; 6 Division of Oral and Maxillofacial Reconstructive Surgery, Department of Disease Management Dentistry, Graduate School of Dentistry, Tohoku University, Sendai 980-8575, Miyagi, Japan; kensuke.yamauchi.a1@tohoku.ac.jp

**Keywords:** oral microbiome, saliva, dental plaque, tongue coating, microbial diversity, systemic factors, 16S rRNA sequencing, functional prediction, oral–systemic interaction

## Abstract

The oral cavity contains several microbial niches, including saliva, dental plaque and tongue coating, each shaped by distinct local environments and host factors. This study compared the ecological and functional characteristics of the microbiomes of these three oral sites within the same individuals and examined host conditions associated with their variation. Saliva, supragingival plaque and tongue coating samples were collected simultaneously from 31 adults without clinical oral lesions. The bacterial 16S rRNA gene (V3–V4 region) was sequenced using the Illumina MiSeq platform, and analyses included α and β diversity, Mantel correlations, differential abundance tests, network analysis and functional prediction. The three sites displayed a clear ecological gradient. Saliva and tongue coating were taxonomically similar but were influenced by different host factors, whereas plaque maintained a distinct, biofilm-like structure with limited systemic influence. Functional divergence was most pronounced on the tongue coating despite its taxonomic similarity to saliva, whereas functional differences between saliva and plaque were modest despite larger taxonomic separation. These findings indicate that microbial composition and function vary independently across oral niches and support the need for multi-site sampling to more accurately characterize oral microbial ecology.

## 1. Introduction

The human oral cavity harbors one of the most diverse and functionally complex microbial ecosystems in the body, comprising distinct habitats such as saliva, plaque, and tongue coating. Each of these microenvironments exhibits unique physicochemical conditions that influence the structure and function of the resident microbiota. Alterations in oral microbial communities have been linked not only to oral diseases such as periodontitis and dental caries but also to systemic disorders, including diabetes mellitus, cardiovascular disease, and respiratory illness [1,2,3]. Thus, the oral microbiome is increasingly recognized as a dynamic interface connecting local and systemic health.

The salivary microbiota is generally regarded as relatively stable over time, reflecting the overall oral microbial status of an individual [4]. In contrast, the tongue coating microbiota often resembles the salivary microbiota and is considered part of a “mucosal-type” microbial continuum [5]. The plaque microbiota, on the other hand, forms dense biofilms with a unique ecological structure and metabolic cooperation distinct from mucosal sites [6]. Despite their biological interconnectedness, few studies have directly compared these three oral niches within the same individuals, leaving unresolved the question of how local and systemic factors collectively shape oral microbial ecosystems.

Recent advances in 16S rRNA gene sequencing and bioinformatics have enabled detailed characterization of both microbial composition and function. Tools such as PICRUSt2 allow prediction of metabolic pathways from amplicon data, providing insight into microbial contributions to carbohydrate metabolism, lipid biosynthesis, and inflammatory regulation [7,8]. In oral environments, these predicted pathways may explain host–microbe interactions in disease processes such as periodontitis, halitosis, and metabolic dysfunction [9]. However, comprehensive comparisons of both taxonomic and functional differences among saliva, plaque, and tongue coating microbiota in the same individuals remain scarce. Moreover, while systemic conditions such as diabetes and hyperlipidemia are known to influence oral microbiota composition [3], it remains unclear whether such effects occur uniformly across oral sites or selectively impact specific microbial habitats. Lifestyle and oral conditions, including alcohol consumption, smoking, oral hygiene, and the number of remaining teeth, have also been associated with microbial diversity [10], but site-specific responses have not been well characterized.

In this study, we investigated the microbiota of saliva, plaque, and tongue coating collected simultaneously from 31 participants without mucosal disease. Using 16S rRNA gene sequencing targeting the V3–V4 region, we compared microbial diversity, inter-site correlations, and predicted functional pathways. We further assessed the influence of systemic, oral, and lifestyle factors to elucidate the ecological structure and host associations of the oral microbiome, providing new insight into the ecological interconnection of distinct microbial communities within the oral cavity.

## 2. Materials and Methods

### 2.1. Study Population

This study included 31 adult participants who visited the Department of Oral and Maxillofacial Surgery, Tohoku University Hospital (Sendai, Japan) for evaluation of suspected oral mucosal disorders between April 2023 and March 2024. All participants were confirmed to be free from mucosal disease and had not received systemic antibiotics or antifungal agents within three months before sampling. Clinical information (age, sex, systemic medical history including diabetes, hypertension, and hyperlipidemia, oral hygiene status, number of teeth, DMFT index, and periodontal parameters) was obtained during the same visit.

### 2.2. Sample Collection

Unstimulated whole saliva was collected in 30 mL sterile tubes (≥1 mL per participant), followed by supragingival dental plaque sampling from the buccal surfaces of molars using sterile curettes and tongue coating sampling by gentle scraping of the dorsal tongue surface, prior to any food intake, tooth brushing, oral rinsing, or any form of treatment. After collection, samples were immediately transferred to the Tohoku University Clinical Biobank for cryogenic storage and quality control [11]. All 31 samples were retrieved together from the biobank and processed as a single batch. The study was approved by the Ethics Committee of the Tohoku University Graduate School of Dentistry (approval no. 25091) and conducted in accordance with the Declaration of Helsinki. Written informed consent was obtained from all participants.

### 2.3. Clinical Parameters and Oral Health Definitions

Clinical oral examinations were performed by staff dentists from the Department of Oral and Maxillofacial Surgery using standard dental mirrors and periodontal probes. The number of remaining teeth was counted excluding third molars. Participants were classified as having periodontitis if 4 mm or deeper periodontal pockets were present in 20% or more of examined teeth, with the threshold determined in reference to the case definitions jointly developed by the American Academy of Periodontology (AAP) and the European Federation of Periodontology (EFP) for grading the severity of periodontal disease [12,13]. Oral hygiene was evaluated using the Plaque Control Record (PCR) method with disclosing solution. Participants with PCR scores < 20% were classified as having good oral hygiene, whereas those with PCR ≥ 20% were classified as having poor oral hygiene [14]. Dental caries experience was evaluated using the DMFT index, representing the total number of Decayed (D), Missing (M), and Filled (F) in according to World Health Organization, Oral Health Surveys: Basic Methods, 5th [15]. All oral examinations and microbial sampling were conducted on the same day.

### 2.4. DNA Extraction and Sequencing

Genomic DNA was extracted using the DNeasy PowerSoil Pro Kit (Qiagen, Hilden, Germany) following the manufacturer’s protocol. This kit incorporates both chemical lysis and bead-beating-based mechanical disruption and is specifically designed to ensure efficient lysis of Gram-positive bacteria with thick cell walls. All saliva, dental plaque and tongue coating samples were processed simultaneously using identical reagents and protocols, with DNA extraction performed individually for each sample tube to minimize technical variation and cross-sample contamination.

The V3–V4 region of the bacterial 16S rRNA gene was amplified using primers 341F (5′-CCTACGGGNGGCWGCAG-3′) and 805R (5′-GACTACHVGGGTATCTAATCC-3′) via a two-step PCR protocol as same as our previous study [16]. The first PCR amplified the target region, and the second PCR added Illumina adapters and barcodes. Amplification was performed with Ex Taq DNA Polymerase (TaKaRa Bio Inc., Kusatsu, Japan). DNA concentrations were measured using a Qubit 2.0 Fluorometer and dsDNA HS Assay Kit (Life Technologies, Carlsbad, CA, USA). Libraries were diluted to 60 pM with 20% PhiX and sequenced on an Illumina MiSeq (2 × 300 bp paired-end, MiSeq Reagent Kit v3; Illumina, San Diego, CA, USA).

To monitor potential contamination, negative controls were included throughout the workflow. Nuclease-free water was incorporated as a negative control from the first PCR step onward and was also included in each sequencing run. These controls consistently showed negligible amplification, indicating the absence of detectable reagent- or laboratory-derived contamination. Therefore, the use of mock community standards was not considered necessary for this study.

### 2.5. Microbiome Analysis

Amplicon data were processed with QIIME 2 (version 2023.2) [17]. Quality filtering and denoising were performed with the DADA2 plugin (version 2022.10) [18], optimized for the V3–V4 region. The first 20 bases corresponding to the primer sequences were removed from both forward and reverse reads, and the remaining reads were truncated at 280 bp (forward) and 240 bp (reverse) before merging. Sequences were aligned using MAFFT (version 7.520) [19], and a phylogenetic tree was constructed with FastTree 2 [20]. Taxonomic classification was conducted using a Naïve Bayes classifier trained on the Greengenes 2 database [21] with a confidence threshold of 0.7.

### 2.6. Diversity Analysis

Alpha diversity was calculated using observed features [17], Shannon entropy [22], and Faith’s phylogenetic diversity (Faith-PD) [23]. Differences among sample types were evaluated using the paired Steel–Dwass test. Beta diversity was computed using weighted and unweighted UniFrac distances [24], visualized by principal coordinate analysis (PCoA), and tested by PERMANOVA [25]. Inter-site similarities (saliva vs. plaque, saliva vs. tongue coating and plaque vs. tongue coating) were evaluated by the Mantel test based on Spearman’s correlation (ρ) without covariate adjustment [26].

### 2.7. Differential Abundance and Functional Prediction

Differentially abundant features were identified using ANCOM-BC [27] in pairwise comparisons across saliva, plaque, and tongue coating. Because species-level resolution is limited in 16S rRNA sequencing, bacterial taxa were analyzed at the genus level, while predicted metabolic pathways were evaluated using PICRUSt2 (version 2.5.2) with MetaCyc pathway annotations [7,8]. Functional β diversity was assessed using Bray–Curtis dissimilarity and visualized by PCoA [28], with statistical significance tested using PERMANOVA. For visualization in 100% stacked bar plots, pathways and class-level taxa with relative abundances < 1% were grouped as “Others”.

### 2.8. Background Factor Analysis and Co-Occurrence Networks

Associations between α and β diversity or the *Firmicutes*/*Bacteroidetes* (F/B) ratio and host factors (e.g., diabetes, drinking habit, oral hygiene, number of teeth, DMFT index) were examined using multivariate regression models. Effect sizes were estimated using the least-squares method and visualized as circle areas on bivariate plots, with red indicating significant (*p* < 0.05) and green indicating marginal (0.05 ≤ *p* < 0.10) associations. Microbial co-occurrence networks were constructed using Spearman’s ρ and visualized in Cytoscape (version 3.10.4) [29]. Edges represented positive correlations (ρ ≥ 0.4, *p* < 0.05) or negative correlation (−0.4 ≥ ρ, *p* < 0.05) and node size reflected relative taxon abundance.

### 2.9. Statistical Analysis

All statistical analyses were performed using QIIME 2, R (version 4.3.1; R Core Team, Vienna, Austria), and JMP Pro (version 17, SAS Institute Inc., Cary, NC, USA). *p* values < 0.05 were considered statistically significant.

## 3. Results

### 3.1. Demographic, Systemic, and Oral Clinical Characteristics of the 31 Participants

A total of 31 participants were included in this study. Their demographic, systemic, and oral health characteristics are summarized in Table 1. The study population consisted of adults with varying systemic conditions, including diabetes and hypertension. The number of remaining teeth and oral hygiene levels also differed among individuals. These data illustrate the heterogeneity of participants’ backgrounds, highlighting the potential influence of both systemic and local oral factors on the microbial composition in the oral cavity.

### 3.2. Comparison of α Diversity Among Saliva, Plaque and Tongue Coating

Three α diversity metrics, observed features, Shannon entropy and Faith’s phylogenetic diversity (Faith-PD), were compared among saliva, plaque and tongue coating from the same 31 participants.

**Figure 1:**
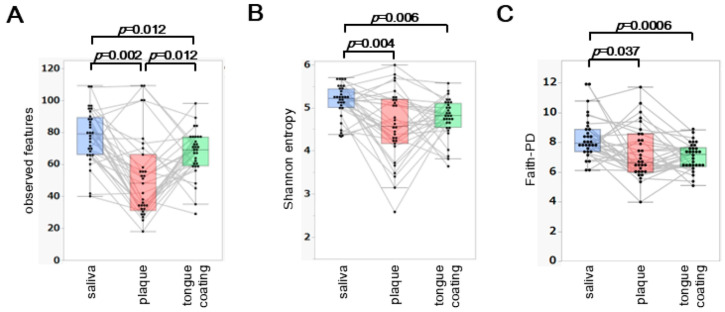
Comparison of α diversity indices among saliva, plaque and tongue coating. (**A**–**C**) Boxplots of observed features (**A**), Shannon entropy (**B**) and Faith’s phylogenetic diversity (Faith-PD) (**C**) across saliva, plaque and tongue coating. Each thin line links samples from the same individual to illustrate intra-individual variation across oral sites. Statistical comparisons were performed using the paired Steel–Dwass test and only significant *p* values (*p* < 0.05) are shown within the figure.

Observed features differed significantly across all three sites (saliva > tongue coating > plaque; *p* < 0.05) (Figure 1A). For Shannon entropy, saliva showed the highest diversity, whereas plaque and tongue coating did not differ significantly (Figure 1B). Faith-PD showed a pattern identical to that of Shannon entropy (Figure 1C), with saliva exhibiting the highest phylogenetic diversity and no significant difference between tongue coating and plaque.

Overall, saliva consistently displayed the highest α diversity, while plaque and tongue coating showed similar diversity profiles. The lower richness in plaque likely reflects its more specialized, biofilm-associated microbial structure compared with the more dynamic environments of saliva and tongue coating.

### 3.3. Background Factors Influencing a Diversity

The associations between host and lifestyle factors and α diversity were analyzed separately for saliva, plaque, and tongue coating. Each circle represents one explanatory variable, with its area corresponding to the effect magnitude in the multivariate model. Red circles denote significant associations (*p* < 0.05), green circles indicate marginal associations (0.05 ≤ *p* < 0.10), and the position along the axis reflects the direction and relative contribution of each factor.

**Figure 2:**
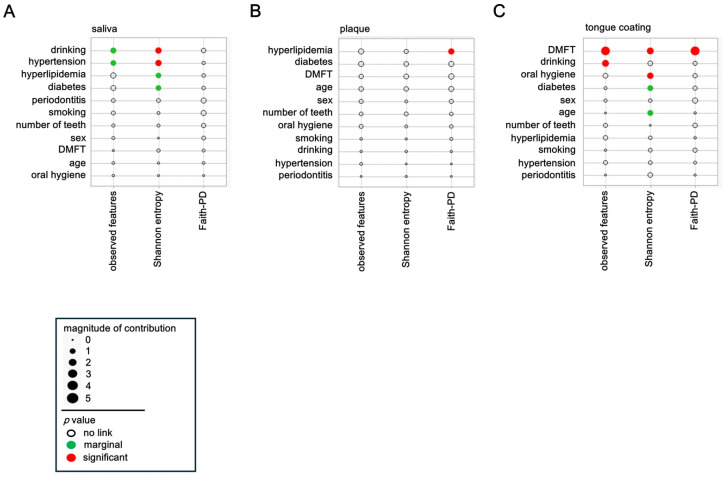
Background factors influencing α diversity. (**A**–**C**) Background factors associated with α diversity in saliva (**A**), plaque (**B**) and tongue coating (**C**). Circle size represents effect magnitude, and circle color indicates *p* value categories (red: *p* < 0.05; green: 0.05 ≤ *p* < 0.10; white: *p* ≥ 0.10). Statistical associations were estimated using the least-squares method.

In saliva, Shannon entropy showed significant associations (red circles) with alcohol consumption and hypertension, indicating that the balance of salivary microbial communities is sensitive to behavioral and vascular factors (Figure 2A). Moreover, observed features also tended to increase (green circles) with alcohol consumption and hypertension, suggesting that both richness and evenness are modulated by these conditions. Diabetes and hyperlipidemia displayed marginal associations (green circles) with α diversity, implying that systemic metabolic health may subtly influence the salivary ecosystem.

In plaque, only Faith-PD diversity showed a significant positive relationship (red circle) with hyperlipidemia, while other systemic factors exerted minimal effects (Figure 2B). This indicates that plaque diversity is primarily governed by local biofilm dynamics, with limited responsiveness to systemic conditions.

In tongue coating, the DMFT index exhibited strong and consistent associations (red circles) across all α diversity metrics, observed features, Shannon entropy, and Faith-PD (Figure 2C), demonstrating that cumulative oral disease experience profoundly shapes tongue surface microbial diversity. Additionally, observed features were marginally associated with alcohol consumption (green circle), and Shannon entropy was significantly influenced by oral hygiene (red circle). Diabetes also showed a marginal association (green circle), suggesting that metabolic disorders may affect the microbial ecology of the tongue dorsum.

Collectively, these findings indicate that systemic and lifestyle-related factors predominantly shape salivary and tongue coating diversity, whereas plaque diversity remains locally constrained. Thus, saliva and tongue coating microbiota appear to be more strongly influenced by systemic and lifestyle-related factors, while plaque is comparatively stable and locally regulated. This gradient of systemic influence across oral sites underscores the complex interplay between host metabolism, behavior, and site-specific microenvironments in regulating oral microbial diversity.

### 3.4. β Diversity Analysis of Saliva, Plaque and Tongue Coating Microbiota

To assess inter-site differences in the overall composition of the oral microbiota, principal coordinate analysis (PCoA) was performed based on weighted and unweighted UniFrac distances. Differences among the three oral sites, saliva, plaque, and tongue coating, were statistically evaluated using permutational multivariate analysis of variance (PERMANOVA).

**Figure 3:**
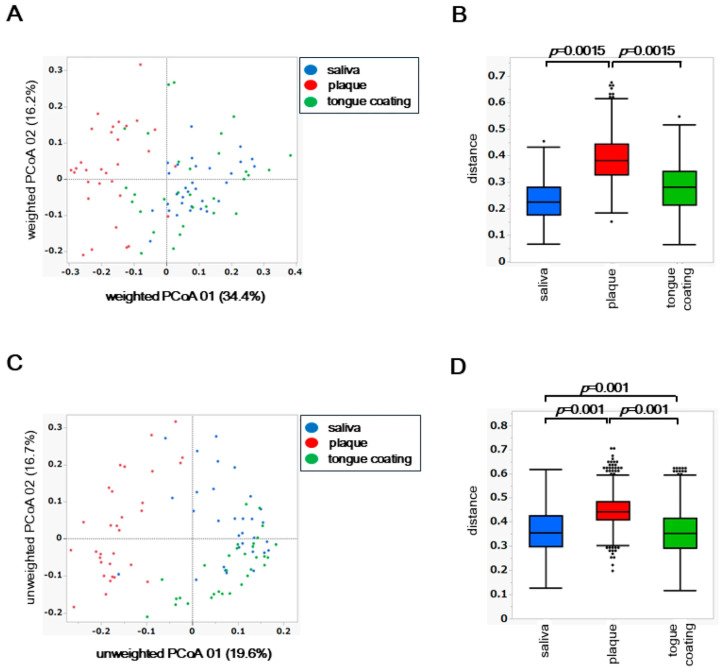
β diversity analysis of saliva, plaque and tongue coating microbiota. (**A**) PCoA plot based on weighted UniFrac distances. (**B**) Boxplot of pairwise weighted UniFrac distances. (**C**) PCoA plot based on unweighted UniFrac distances. (**D**) Boxplot of pairwise unweighted UniFrac distances. Statistical analyses were performed using PERMANOVA, and only *p* values < 0.05 are shown within each figure.

In the weighted UniFrac PCoA, which reflects differences in the abundance of dominant taxa, the microbial community structure of plaque was clearly separated from those of saliva and tongue coating (Figure 3A). This finding indicates that plaque harbors a distinct microbiome shaped by its dense and adherent biofilm environment, where abundant taxa dominate in a site-specific manner. PERMANOVA confirmed a significant difference among the three oral sites (*p* < 0.05), primarily driven by the compositional divergence of plaque from the other two niches (Figure 3B).

In contrast, the unweighted UniFrac PCoA, which is based on the presence or absence of taxa including low-abundance species, revealed that saliva, plaque, and tongue coating each formed distinct clusters (Figure 3C). PERMANOVA of the unweighted distances also showed significant differences among all site pairs (*p* < 0.05), suggesting that rare or low-frequency taxa further contribute to the differentiation among oral niches (Figure 3D).

Together, these findings demonstrate that while the dominant bacterial community of plaque differs most strongly from saliva and tongue coating, the entire microbial assemblage, including rare taxa, differs across all oral sites. This indicates a layered ecological organization in the oral cavity, in which abundant core microbiota are partially shared between saliva and tongue coating, whereas niche-specific and rare species define finer structural distinctions.

### 3.5. Background Factors Associated with β Diversity

To examine how host background factors shape overall microbial community structure, associations between each variable and β diversity were evaluated using both weighted and unweighted UniFrac PCoA results for saliva, plaque and tongue coating. Weighted analyses represent variations driven by dominant taxa, whereas unweighted analyses highlight differences in rare or minor taxa.

**Figure 4:**
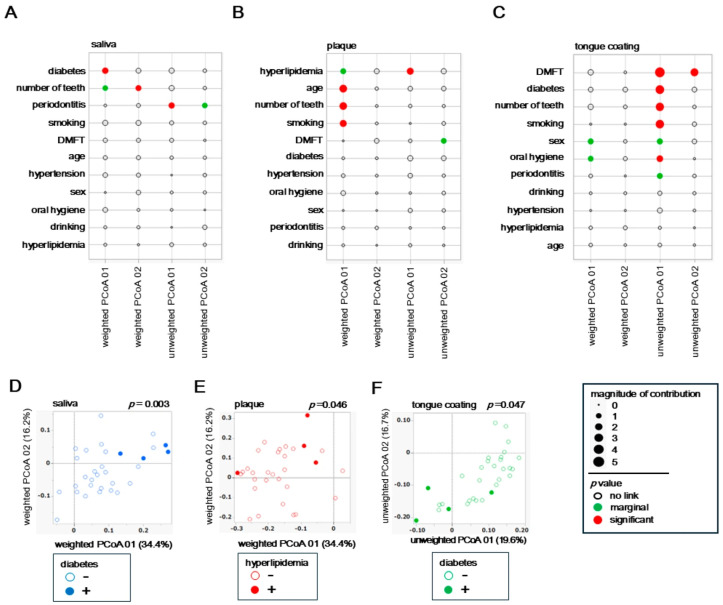
Background factors associated with β diversity. (**A**–**C**) Relative contribution of background variables to weighted and unweighted UniFrac PCoA axes in saliva (**A**), plaque (**B**) and tongue coating (**C**). Circle size represents effect magnitude, and circle color indicates *p* value categories (red: *p* < 0.05; green: 0.05 ≤ *p* < 0.10; white: *p* ≥ 0.10). Statistical associations were estimated using the least-squares method. (**D**–**F**) UniFrac PCoA plots stratified by selected background factors. In all panels, circle color indicates the sample type (blue: saliva; red: plaque; green: tongue coating). Filled circles represent participants with the condition, and outlined circles represent those without the condition. (**D**) Saliva (weighted UniFrac): diabetes status. (**E**) Plaque (weighted UniFrac): hyperlipidemia status. (**F**) Tongue coating (unweighted UniFrac): diabetes status. Statistical analyses were performed using PERMANOVA, and *p* values are shown within each figure.

In saliva, the weighted PCoA showed strong associations with diabetes and number of teeth (Figure 4A), whereas the unweighted PCoA indicated a closer relationship with periodontitis. In plaque, the weighted PCoA revealed involvement of multiple factors such as hyperlipidemia, age, number of teeth and smoking history (Figure 4B), while the unweighted PCoA identified hyperlipidemia as the sole contributing factor. In tongue coating, the weighted analysis suggested potential influences of sex and oral hygiene (Figure 4C), whereas the unweighted PCoA indicated broader associations involving diabetes, various oral clinical indices, and smoking. These results indicate that the factors influencing microbial β diversity differed markedly among oral sites, with each niche being affected by distinct systemic or oral conditions. For example, systemic metabolic conditions predominantly affected the salivary microbiota, both systemic and lifestyle factors interacted within plaque, and complex oral environmental conditions exerted the strongest effects on the tongue coating community.

To further illustrate these associations, PCoA plots were generated for the most influential systemic factors identified in each site. In saliva, individuals with diabetes (blue dots) were clearly separated along the weighted PCoA axis (Figure 4D), confirming that diabetes significantly alters dominant salivary bacterial composition. In plaque, subjects with hyperlipidemia (red dots) showed partial separation in the weighted PCoA (Figure 4E), suggesting that lipid metabolism influences biofilm-associated microbiota. In tongue coating, participants with diabetes (green dots) were separated in the unweighted PCoA, indicating compositional differences among low-abundance taxa on the tongue surface.

Together, these results demonstrate a layered pattern of host–microbiome interaction. Systemic conditions primarily reshape the dominant taxa composition in saliva and plaque, while intraoral and metabolic factors modulate the presence of minor taxa in tongue coating. Thus, saliva and tongue coating represent more dynamic and host-responsive niches, whereas plaque remains ecologically stable and locally constrained.

### 3.6. Correlation of β Diversity Among Oral Sites Based on Mantel Test Analysis

**Figure 5:**
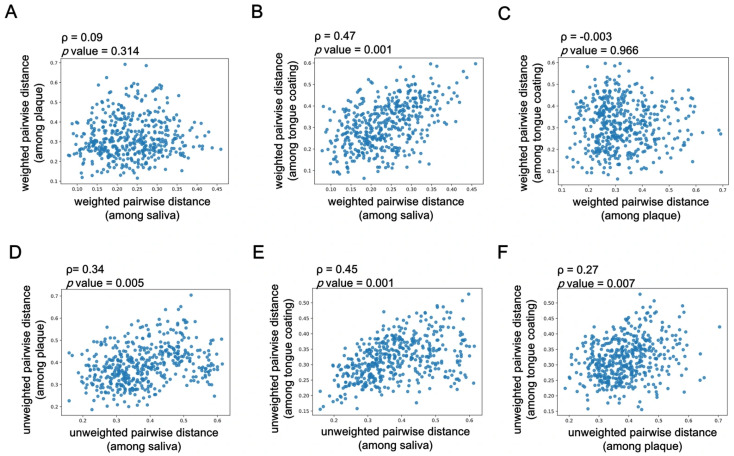
Correlation of β diversity among oral sites based on Mantel test analysis. (**A**–**C**) Pairwise correlations based on weighted UniFrac distance matrices, calculated using the Mantel test: (**A**) saliva vs. plaque, (**B**) saliva vs. tongue coating and (**C**) plaque vs. tongue coating. (**D**–**F**) Pairwise correlations based on unweighted UniFrac distance matrices, also assessed using the Mantel test: (**D**) saliva vs. plaque, (**E**) saliva vs. tongue coating and (**F**) plaque vs. tongue coating. Each scatterplot represents the relationship between two oral sites, with Spearman’s correlation coefficient (ρ) and the corresponding *p* values shown within each figure.

After identifying how systemic, lifestyle, and oral factors influenced β diversity at each oral site (Figure 4), we next examined how similar the β diversity patterns were among saliva, plaque, and tongue coating within the same individuals using Mantel correlation analysis. Figure 5A–C show correlations based on weighted UniFrac distances, while Figure 5D–F depict those based on unweighted UniFrac distances, using samples collected simultaneously from the same individuals.

In Figure 5A, Spearman’s correlation coefficient (ρ) between saliva and plaque was weak and not statistically significant (ρ = 0.09, *p* = 0.314), suggesting that salivary microbiota composition differs considerably from that of the plaque biofilm. In contrast, Figure 5B shows that saliva and tongue coating exhibited the strongest correlation (ρ = 0.47, *p* = 0.001), indicating that these two microbial niches share highly similar community structures. Figure 5C, representing plaque versus tongue coating, showed almost no correlation (ρ = −0.003, *p* = 0.966), reflecting the distinct microbial profiles between the hard biofilm and the mucosal surface.

Similarly, the unweighted UniFrac-based correlations (Figure 5D–F) showed consistent tendencies. Figure 5D (saliva vs. plaque) showed a moderate but weaker correlation (ρ = 0.34, *p* = 0.005), while Figure 5E (saliva vs. tongue coating) again showed the highest correlation (ρ = 0.45, *p* = 0.001). Figure 5F (plaque vs. tongue coating) exhibited a lower but still significant association (ρ = 0.27, *p* = 0.007), suggesting a limited overlap of minor taxa between these two sites.

Overall, these Mantel test results demonstrate that saliva and tongue coating harbor the most similar microbial community structures, whereas plaque remains compositionally distinct and locally stable. This pattern highlights the ecological heterogeneity within the oral cavity, saliva and tongue coating, reflecting dynamic and interchangeable communities, while plaque represents a more static, site-specific biofilm ecosystem.

### 3.7. Class-Level Composition and Factors Associated with the Firmicutes/Bacteroidetes (F/B) Ratio

At the class level, microbial compositions differed markedly among saliva, plaque, and tongue coating, with only classes showing a relative abundance ≥ 1% displayed and taxa below this threshold were grouped as “Others”.

**Figure 6:**
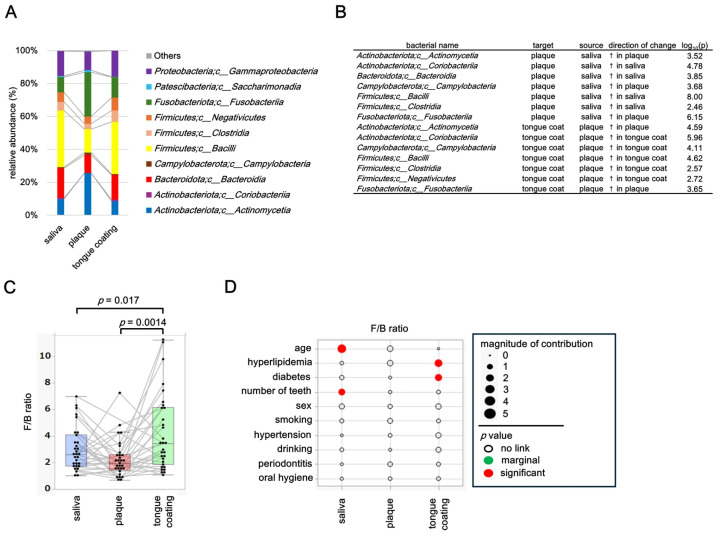
Class-level composition and factors associated with the *Firmicutes/Bacteroidetes* (F/B) ratio. (**A**) Stacked bar plot showing the relative abundance of bacterial classes across oral sites (taxa with <1% abundance grouped as “Others”). (**B**) Table showing significant pairwise differences (*p* < 0.05) in class-level relative abundance excluding classes categorized as “Others”, identified using the Steel–Dwass test. Arrows (↑) indicate the oral site (saliva, plaque or tongue coating) in which the relative abundance of the corresponding bacterial class was significantly higher in the pairwise comparison. (**C**) Boxplot of the *Firmicutes*/*Bacteroidetes* ratio across saliva, plaque and tongue coating; thin lines connect samples from the same individual. Only *p* values < 0.05 are shown within the figure. (**D**) Background factors associated with the F/B ratio. Circle size indicates effect magnitude and circle color indicates *p* value categories (red: *p* < 0.05; green: 0.05 ≤ *p* < 0.10; white: *p* ≥ 0.10). Statistical associations were estimated using the least-squares method.

Saliva and tongue coating were dominated by *Bacilli* and *Bacteroidia*, whereas plaque exhibited higher proportions of *Actinobacteria* and *Fusobacteria*, reflecting the anaerobic and biofilm-enriched nature of this niche (Figure 6A). Pairwise comparisons using the Steel–Dwass test identified several classes with significant inter-site differences (Figure 6B). *Bacilli* and *Bacteroidia* were significantly more abundant in saliva and tongue coating, while *Actinobacteria* and *Fusobacteria* were enriched in plaque, highlighting ecological differentiation between fluid-associated and surface-associated habitats in the oral cavity.

The *Firmicutes*/*Bacteroidetes* (F/B) ratio, an indicator of microbial balance, varied significantly among oral sites (Figure 6C). The ratio was highest in tongue coating, lowest in plaque, and intermediate in saliva (*p* = 0.017 for saliva vs. plaque and *p* = 0.0014 for saliva vs. tongue coating, Steel–Dwass test). Each thin line in the boxplot connects samples from the same participant, showing consistent intra-individual patterns across oral sites. Background factors influencing the F/B ratio were examined using multivariate models for each site (Figure 6D). In saliva, the F/B ratio was significantly associated with age and number of teeth, suggesting that oral aging and tooth loss influence microbial balance in salivary communities. In plaque, no significant factors were detected, indicating that the plaque microbiome is locally regulated and less influenced by systemic background. In tongue coating, the F/B ratio was significantly associated with diabetes and hyperlipidemia, suggesting that microbial balance on the tongue dorsum reflects systemic metabolic status.

These results demonstrate that both microbial composition and the F/B ratio differ substantially among oral sites. While salivary F/B ratios are affected by oral conditions such as age and tooth loss, tongue coating F/B ratios are modulated by systemic metabolic disorders including diabetes and hyperlipidemia. In contrast, the plaque microbiome remains stable and locally maintained. Together, these findings indicate that saliva and tongue coating are sensitive indicators of oral and systemic health, whereas plaque represents a more resilient and localized microbial ecosystem.

### 3.8. Class-Level Heatmap Analysis of Oral Microbiota

To further compare the microbial community structures among saliva, plaque, and tongue coating, a heatmap based on the relative abundance of bacterial classes was generated. Hierarchical clustering revealed distinct compositional patterns among the three oral sites.

**Figure 7:**
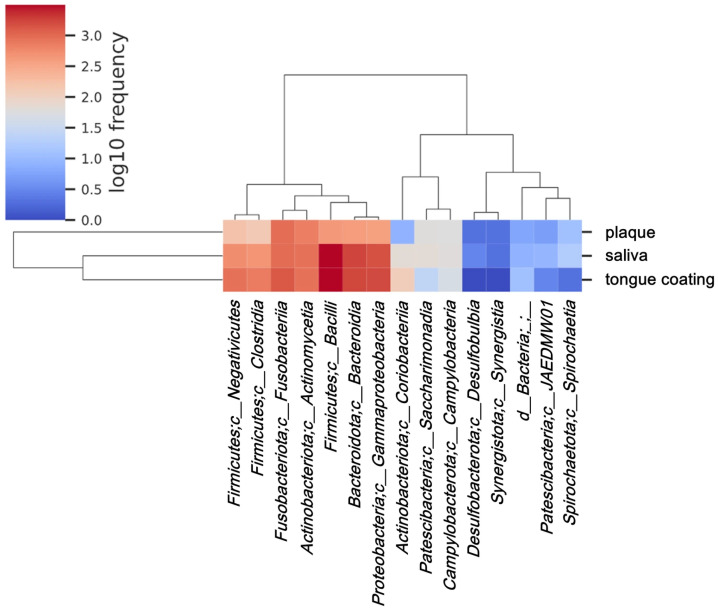
Class-level heatmap analysis of oral microbiota. Heatmap of the log_10_-transformed frequencies of bacterial classes in saliva, plaque and tongue coating. Values were averaged within each sample type before plotting. Hierarchical clustering was performed using Euclidean distance and Ward’s linkage method to group classes with similar distribution patterns. Sample types are shown on the horizontal axis, and bacterial classes are shown on the vertical axis.

Saliva and tongue coating samples clustered together, indicating that these two habitats share a closely related class-level microbiota (Figure 7). Both were characterized by the dominance of *Bacilli* and *Bacteroidia*, representing mucosal-type bacterial communities that are continuously exposed to oxygen. In contrast, plaque samples formed a distinct cluster, enriched in Actinobacteria and Fusobacteria, which are typical of anaerobic, biofilm-associated environments. This clustering pattern is consistent not only with theα diversity findings (Figure 1 and Figure 2) but also with the β diversity analyses (Figure 3, Figure 4 and Figure 5), which showed that saliva and tongue coating have similar overall microbial structures. These results indicate that saliva and tongue coating harbor a related and dynamically connected microbiota, whereas plaque constitutes an ecologically distinct and more stable microbial community.

### 3.9. Correlation Network Analysis of Class-Level Bacteria Within and Across Oral Sites

To explore intra- and inter-site relationships among bacterial taxa, correlation networks were constructed using Spearman’s correlations (ρ ≥ 0.4, *p* < 0.05). Each node represents a bacterial class, node size corresponds to its relative abundance, and each edge indicates a significant positive correlation (red) or negative collection (blue).

**Figure 8:**
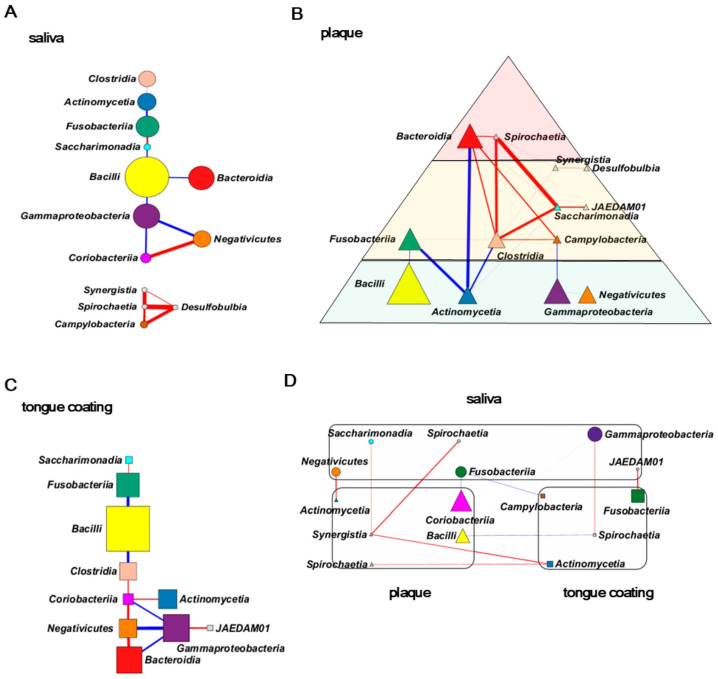
Correlation network analysis of class-level bacteria within and across oral sites. Correlation networks were constructed using Spearman correlations (ρ ≥ 0.4, *p* < 0.05). Nodes represent bacterial classes; node size reflects their relative abundance within the corresponding sample type, and node shape denotes the oral site (circle: saliva; triangle: plaque; square: tongue coating). Edge colors indicate the direction of correlation (red: positive; blue: negative), and edge thickness represents correlation strength. Node colors correspond to the class-level color scheme used in Figure 7. (**A**) Network of saliva samples. (**B**) Network of plaque samples displayed over the simplified Socransky complex pyramid. (**C**) Network of tongue coating samples. (**D**) Cross-site network integrating saliva, plaque and tongue coating; node size reflects the relative abundance within each respective site.

In saliva, the network showed a simple and linear pathway, connecting a few major taxa such as *Bacilli* and *Bacteroidia* (Figure 8A). This structure indicates a relatively stable and streamlined microbial community in which a small number of dominant taxa form a continuous relationship network.

The plaque network was the most complex, forming a dense web of interconnections consistent with the biofilm nature of plaque (Figure 8B). It was overlaid on the Socransky pyramid, a classic model of subgingival microbial ecology [30]. Even at the class level, the hierarchical structure resembled this model, where basal taxa (e.g., *Actinobacteria*) appeared to stabilize or suppress upper-layer taxa (e.g., *Fusobacteria*), reflecting ecological control within the plaque biofilm. The tongue coating network was also simple, similar to saliva, and largely followed a single main route of connections among *Bacteroidia* and *Negativicutes* (Figure 8C). This linear topology suggests that the tongue surface microbiota forms a stable and less complex community under dynamic but controlled mucosal conditions. Figure 8D integrates the three oral sites into a cross-site correlation network. No significant edges were detected between identical bacterial classes across different sites. Instead, multiple significant correlations emerged between distinct classes across sites, implying interdependent but functionally non-redundant relationships among oral habitats. These cross-site links may reflect ecological accelerators or brakes that modulate microbial balance and adaptability within the oral ecosystem, a novel finding unique to this intra-individual design.

Overall, saliva and tongue coating shared simple, nearly linear network structures, whereas plaque displayed a complex hierarchical pattern reminiscent of the Socransky pyramid. The integrated network revealed unique inter-class relationships rather than direct overlaps, suggesting that oral microbial communities across different niches are ecologically connected yet functionally distinct within the same individual.

### 3.10. ANCOM-BC Analysis of Differential Bacterial Genus Abundance Across Oral Sites

**Figure 9:**
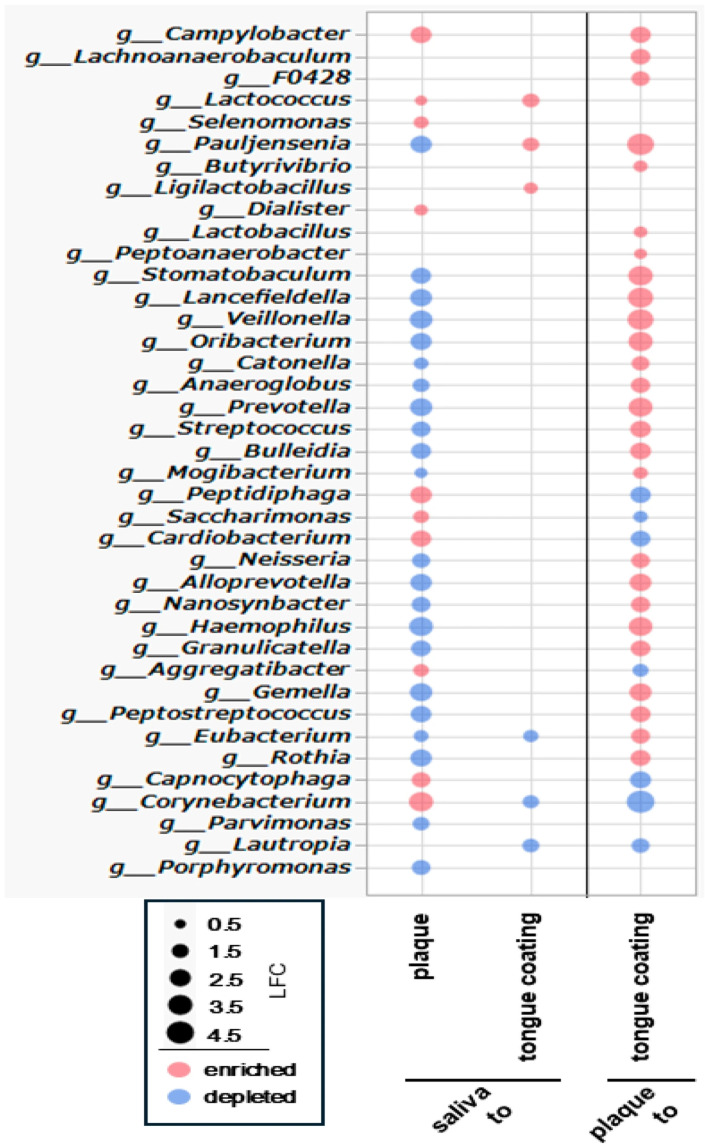
ANCOM-BC analysis of differential bacterial genus abundance across oral sites. Differentially abundant bacterial genera among saliva, plaque and tongue coating were identified using ANCOM-BC and visualized in a bubble plot. Each bubble represents a genus with significant differences (*q* < 0.05). Bubble color indicates the direction of log_2_ fold change (LFC) (red: enriched; blue: depleted), and bubble size reflects the magnitude of the log_2_ fold change. Comparisons are shown for saliva → plaque, saliva → tongue coating and plaque → tongue coating.

To clarify genus-level differences among oral sites, ANCOM-BC was applied to all pairwise comparisons (saliva–plaque, saliva–tongue coating and plaque–tongue coating). Because 16S rRNA sequencing offers limited species-level resolution, analyses were performed at the genus level and visualized in a single bubble plot summarizing significant log_2_ fold changes (Figure 9).

In the comparison between saliva and plaque, plaque exhibited larger divergence, with 10 genera enriched and 22 depleted relative to saliva. Enriched genera included anaerobic and biofilm-associated taxa such as Campylobacter, Lactococcus, and Selenomonas, whereas genera that are more abundant and commonly found in saliva such as Streptococcus and Neisseria were depleted. These findings highlight the strong ecological separation between fluid-associated (saliva) and biofilm-associated (plaque) communities.

In contrast, the comparison between saliva and tongue coating revealed only modest differences, with 3 genera enriched and 3 depleted. Enriched taxa included mucosa-associated genera such as Lactococcus, reflecting the close ecological relationship between saliva and the tongue coating and their shared exposure to oxygenated mucosal surfaces.

A broader shift emerged in the comparison between plaque and tongue coating, in which tongue coating demonstrated 26 enriched and 7 depleted genera. Mucosal or oxygen-tolerant genera such as *Streptococcus*, *Neisseria*, and *Haemophilus* were enriched, whereas several plaque-associated anaerobic taxa were reduced. This pattern underscores the distinction between the dense anaerobic biofilm of plaque and the more dynamic, oxygen-exposed microbiota of the tongue surface.

In summary, the genus-level ANCOM-BC analysis demonstrates that plaque is the most compositionally distinct niche, while saliva and tongue coating are similar, differing only in a few genera. These findings provide a taxonomic framework that supports the ecological and functional contrasts observed across oral sites in subsequent analyses.

### 3.11. Functional Prediction of Oral Microbiota Using PICRUSt2

Functional prediction of the oral microbiota was performed using PICRUSt2, and Bray–Curtis PCoA was applied to assess differences in predicted metabolic functions among saliva, plaque, and tongue coating.

**Figure 10:**
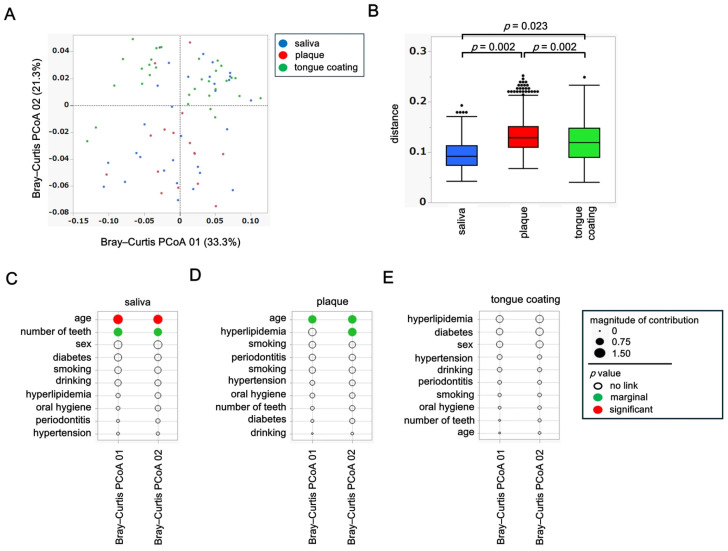
Functional prediction of oral microbiota among saliva, plaque, and tongue coating using PICRUSt2. (**A**) PCoA plot based on Bray–Curtis dissimilarity. (**B**) Boxplot of Bray–Curtis distances among the three oral sites. *p* values with PERMANOVA are shown within the figure. (**C**–**E**) Background factors associated with Bray–Curtis PCoA axes for saliva (**C**), plaque (**D**) and tongue coating (**E**). Circle size represents effect magnitude, and circle color indicates *p* value categories (red: *p* < 0.05; green: 0.05 ≤ *p* < 0.10; white: *p* ≥ 0.10). Statistical associations were estimated using the least-squares method.

The overall ordination pattern showed clear separation among the three sites, similar to the unweighted UniFrac PCoA results (Figure 10A), indicating that differences in minor (low-abundance) taxa substantially contribute to functional divergence across oral habitats. Plaque exhibited the most distinct functional profile (Figure 10B). This suggests that functional differentiation within the oral microbiota arises largely from variation in low-abundance bacterial members.

Background factor analysis revealed that different host characteristics influenced metabolic pathway variation across sites, the functional profile in saliva was significantly associated with age and number of teeth (Figure 10C), implying that oral functional diversity reflects both aging and dentition status. In plaque, age and hyperlipidemia were the main factors associated with Bray–Curtis dissimilarities (Figure 10D), suggesting that biofilm functional capacity is affected by systemic lipid metabolism as well as local aging. In tongue coating, the functional profiles appeared relatively stable (Figure 10E), showing minimal influence from host background factors, consistent with its role as a mucosal surface maintaining ecological homeostasis.

These findings indicate that functional differences among oral sites are primarily driven by minor taxa, and that host background factors such as age, dentition, and lipid metabolism selectively influence site-specific functional profiles.

Saliva reflects age- and dentition-related changes, plaque functions respond to systemic lipid status, and tongue coating functions remain stable within the oral ecosystem.

### 3.12. Differential Analysis of Metabolic Pathways Among Oral Sites

**Figure 11:**
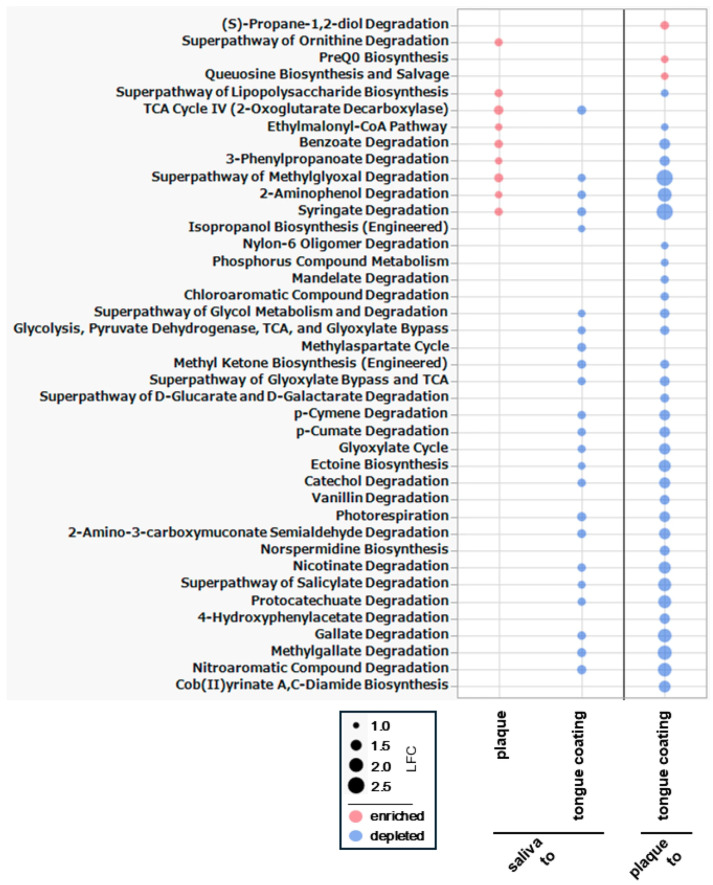
ANCOM-BC analysis of differential metabolic pathways among oral sites. Metabolic pathways predicted by PICRUSt2 were compared across saliva, plaque, and tongue coating using ANCOM-BC. Only pathways showing significant differences (*q* < 0.05) and with an absolute log_2_ fold change (|LFC|) ≥ 1.0 are displayed. Each bubble represents one pathway; red indicates enrichment and blue indicates depletion, and bubble size corresponds to the magnitude of the LFC.

To identify statistically significant functional differences among oral sites, we performed differential abundance analysis of PICRUSt2-inferred metabolic pathways using ANCOM-BC (Figure 11).

In the saliva–plaque comparison, nine pathways were enriched in plaque. LPS biosynthesis was the only increasing biosynthetic pathway, while the other enriched pathways belonged to degradation-related functions. Despite clear genus-level differences, the modest number of pathway shifts suggests partial functional overlap between saliva and plaque.

A greater functional divergence appeared between saliva and tongue coating, where 23 significantly altered pathways were decreased in tongue coating. Many were degradation pathways, including several nitrogen-related degradation pathways. Because these pathways participate in the breakdown of nitrogenous compounds linked to volatile sulfur compound formation, the reductions suggest a distinct metabolic environment on the tongue surface. This functional separation contrasts with the strong taxonomic similarity between saliva and tongue coating.

The plaque–tongue coating comparison showed the widest set of changes, with 37 pathways differing significantly (three enriched and 34 depleted in tongue coating). Many of the depleted pathways were general degradation and nitrogen-related degradation pathways, indicating broad functional down-regulation on the tongue surface. This pattern shows that the tongue coating has a markedly reduced metabolic degradation capacity, making it functionally more distant from plaque as well as from saliva.

Overall, pathway-level ANCOM-BC identifies tongue coating as the most functionally distinct niche, exhibiting the largest number and magnitude of pathway shifts, particularly reductions in degradation and nitrogen-related processes. This overall pattern is consistent with the Bray–Curtis functional separation in Figure 10A,B. However, it clearly contrasts with the genus-level ANCOM-BC results in Figure 9, where saliva and tongue coating appeared taxonomically similar. These findings indicate that tongue coating can display substantial functional divergence even when its genus-level composition overlaps with that of other oral sites.

## 4. Discussion

This intra-individual, multi-site analysis of the oral microbiome revealed a structured ecological gradient across saliva, plaque, and tongue coating. Saliva and tongue coating formed a mucosal-type continuum: they clustered together in α and βdiversity analyses, shared similar class-level compositions, and exhibited simpler network topologies. Plaque, in contrast, remained the most taxonomically and structurally distinct niche, reflecting its biofilm-associated architecture enriched in Actinobacteria and Fusobacteria. Importantly, our analyses demonstrated that taxonomic similarity did not necessarily translate into functional similarity. Although saliva and tongue coating were highly similar at the genus level (Figure 9), pathway-level analyses showed that tongue coating displayed the strongest functional divergence, particularly marked reductions in degradation and nitrogen-related pathways. This finding highlights a decoupling between microbial composition and metabolic potential across oral habitats.

The divergent ecological behavior of these sites can be explained by their structural environments. Plaque represents a mature, matrix-embedded biofilm with low shear stress, steep oxygen gradients, and abundant extracellular polymeric substances that facilitate interspecies cooperation and metabolic stability [31,32]. This structure provides ecological autonomy and explains its limited responsiveness to systemic conditions and its stable biosynthetic functional profile. In contrast, saliva and the tongue surface are dynamic mucosal environments exposed to continuous flow, epithelial turnover, immune molecules, and fluctuating nutrient conditions [5,33]. These characteristics make them more sensitive to systemic metabolic status, including diabetes and hypertension, and to behavioral influences such as alcohol use. In addition, saliva may act as a physical sink for bacteria shed from oral surfaces, particularly the tongue dorsum, due to continuous mechanical abrasion and epithelial turnover. Such mechanical shedding likely contributes to the observed taxonomic similarity between saliva and tongue coating. However, despite this compositional overlap, our pathway-level analyses revealed marked functional divergence, indicating that tongue coating represents a distinct ecological niche with unique metabolic characteristics rather than merely a passive source of salivary bacteria. Prior studies emphasize that physicochemical gradients, including oxygen tension, pH, shear forces, and nutrient flux, shape oral microbial biogeography [2,3,33], and our findings are consistent with this niche-specific framework.

Our β diversity analyses further showed that host factors influenced each site differently. In saliva, systemic conditions such as diabetes and hypertension primarily affected weighted UniFrac axes, suggesting modulation of dominant taxa. Tongue coating, however, was shaped by both metabolic conditions and oral factors such as DMFT and oral hygiene, with pronounced effects on rare taxa in the unweighted analyses. Plaque was influenced minimally by systemic or behavioral conditions, which aligns with the stability expected of a mature biofilm. These observations are consistent with previous reports demonstrating that systemic metabolic disorders, including diabetes and hyperlipidemia, are associated with alterations in oral microbiome composition; however, our results further refine this concept by showing that such systemic influences manifest in a site-specific manner within the oral cavity. These findings underscore that the host factors influencing β diversity differ substantially across sites, and that saliva alone is not sufficient to capture the full ecological heterogeneity of the oral cavity.

Functional prediction provided additional insight into these patterns. It should be noted, however, that functional profiles inferred from 16S rRNA gene sequencing using PICRUSt2 represent predicted metabolic potential based on taxonomic composition and reference genome databases, rather than direct measurements of gene expression or enzymatic activity. Consequently, functional inference is constrained by taxonomic resolution, genome annotation completeness, and inference algorithms, and apparent functional divergence should not be interpreted as true independence from taxonomy. Although plaque was the most taxonomically distinct niche, the largest functional divergence occurred on the tongue surface. Between saliva and tongue coating, 23 pathways were significantly depleted in tongue coating, including many involved in general degradation and nitrogen-related degradation. Between saliva and plaque, only nine pathways differed, mostly involving increased lipopolysaccharide biosynthesis and several degradation pathways in plaque. Comparisons between plaque and tongue coating revealed 37 pathway differences, with tongue coating showing widespread reductions in degradation and nitrogen-related processes. This pattern was consistent with the Bray–Curtis functional separation observed in Figure 10 but contrasted sharply with the genus-level ANCOM-BC findings in Figure 9, where saliva and tongue coating appeared compositionally similar. Thus, tongue coating displayed substantial functional divergence despite limited taxonomic differences, indicating that pathway-level differentiation across oral sites is driven by subtle shifts in minor taxa rather than major compositional changes. Collectively, these findings suggest that systemic metabolic conditions do not uniformly affect all oral habitats but rather interact with local ecological contexts to produce distinct functional signatures across saliva, plaque, and tongue coating.

From a clinical perspective, these results emphasize the importance of assessing microbiota from multiple oral sites rather than relying solely on saliva. Saliva and tongue coating reflected systemic metabolic status and lifestyle habits, while plaque represented a stable, site-specific biofilm ecosystem that captured local disease history such as caries and periodontitis. Because the participants in this study were predominantly older adults (mean age, 60.5 years), caution is warranted when extrapolating these findings directly to younger populations; however, this age group is particularly relevant, as systemic metabolic disorders and lifestyle-related diseases typically emerge or become clinically apparent during middle to older adulthood. Given the growing recognition of oral–systemic connections, including links between diabetes, periodontal disease, and cardiovascular risk [10,34], incorporating tongue-coating assessment into oral microbiome profiling may support earlier detection of systemic metabolic disturbances. The association between DMFT and the tongue-coating microbiome also suggests that the tongue surface may serve as an additional marker for oral disease burden.

In terms of therapeutic implications, effective plaque control continues to depend on mechanical strategies such as toothbrushing, scaling, and interdental cleaning. These methods are required because plaque biofilms are structurally resilient. In contrast, salivary and tongue-coating microbiota may respond more readily to systemic or behavioral interventions, including improved metabolic control, reduced alcohol consumption, reinforcement of oral hygiene, and microbiome-modulating strategies such as probiotics or prebiotics [34,35]. The functional evidence that minor taxa disproportionately shape metabolic potential suggests that future microbiome-based therapies may benefit from targeting microbial functions rather than focusing only on dominant genera.

Strengths of this study include the intra-individual sampling of three distinct oral niches, integration of taxonomic and functional analyses, and combined use of diversity, network, and β diversity approaches. Key limitations include the cross-sectional design, reliance on 16S-based functional inference rather than shotgun metagenomics or metatranscriptomics, and a modest sample size of 31 participants. Future studies should incorporate longitudinal sampling, multi-omics analyses, and systemic biomarkers to clarify causal pathways linking systemic health, oral conditions, and niche-specific microbial functions.

## 5. Conclusions

This study shows that saliva, plaque, and tongue coating differ in both microbial composition and functional potential. Although saliva and tongue coating were taxonomically similar, tongue coating exhibited the most pronounced functional divergence, while plaque remained a stable and distinct biofilm niche. Host factors affected each oral site differently, indicating that sampling multiple sites is important for understanding the full picture of oral microbial ecology and its links to both systemic and oral health.

## Figures and Tables

**Table 1:** Demographic, systemic, and oral clinical characteristics of the 31 participants.

Variable	Value
demographic characteristics	
age, mean ± SD	60.5 ± 11.3
sex, *n* (%)	male 12 (38.7%), female 19 (61.3%)
systemic conditions, *n* (%)	
hypertension	without 19 (61.3%), with 12 (38.7%)
hyperlipidemia	without 27 (87.1%), with 4 (12.9%)
diabetes	without 27 (87.1%), with 4 (12.9%)
lifestyle factors, *n* (%)	
current smoking	without 25 (80.6%), with 6 (19.4%)
drinking habit	without 18 (58.1%), with 13 (41.9%)
dental status, mean ± SD	
number of teeth	23.6 ± 5.5
DMFT	15.5 ± 7.0
DT	0.7 ± 0.9
MT	4.4 ± 5.6
FT	10.5 ± 5.5
oral hygiene and periodontal status, *n* (%)	
oral hygiene	good 9 (29.0%), not good 22 (71.0%)
periodontitis	without 17 (54.8%), with 14 (45.2%)

Systemic conditions (hypertension, hyperlipidemia, diabetes), lifestyle factors (current smoking and drinking), dental status (number of teeth, DMFT, DT, MT, FT), oral hygiene status and periodontitis status are listed. Continuous variables are presented as mean ± standard deviation (SD), and categorical variables as n (%). Abbreviations: DMFT, total number of decayed, missing and filled teeth; DT, decayed teeth; MT, missing teeth; FT, filled teeth. Oral hygiene was categorized as “good” when the plaque control record was <20%. Periodontitis was defined as having periodontal pockets ≥ 4 mm in ≥20% of teeth. These variables were included as covariates in the analyses of α diversity, β diversity and microbial composition.

## Data Availability

The data presented in this study are available on request from the corresponding author. The data are not publicly available due to due to the included personal patient information.
